# Gender Differences in the Social Motivation and Friendship Experiences of Autistic and Non-autistic Adolescents

**DOI:** 10.1007/s10803-015-2669-1

**Published:** 2015-12-23

**Authors:** Felicity Sedgewick, Vivian Hill, Rhiannon Yates, Leanne Pickering, Elizabeth Pellicano

**Affiliations:** Centre for Research in Autism and Education (CRAE), Department of Psychology and Human Development, UCL Institute of Education, University College London, 55-59 Gordon Square, London, WC1H 0NU UK; School of Psychology, University of Western Australia, Perth, Australia

**Keywords:** Autism, Gender, Girls, Friendship, Peer relationships, Social motivation

## Abstract

This mixed-methods study examined gender differences in the social motivation and friendship experiences of adolescent boys and girls with autism relative to those without autism, all educated within special education settings. Autistic girls showed similar social motivation and friendship quality to non-autistic girls, while autistic boys reported having both qualitatively different friendships and less motivation for social contact relative to boys without autism *and* to girls with and without autism. Semi-structured interviews with the adolescents corroborated these findings, with one exception: autistic girls reported high levels of relational aggression within their friendships, suggesting that girls on the autism spectrum in particular may struggle with identifying and dealing with conflict in their social lives.

## Introduction

One of the hallmarks of autism is often-profound difficulties in making and maintaining friendships and understanding social relationships—a feature that has remained prominent in the revised diagnostic criteria for autism, the DSM-5 (APA 2013). As a result, there is a common perception that many autistic^1^ children, young people and adults do not *want* to have friends. Anecdotal reports and increasing empirical evidence suggests, however, that this is not always the case. Children and young people with autism report having friends and best friends (Bauminger et al. 2008) and have a desire to play with, and chat to, their neurotypical peers (Sigman and Ruskin 1999).

This motivation for social relationships was highlighted in recent work by Calder et al. (2013). They studied in-depth the friendship experiences of 12 autistic children in nine London mainstream schools, interviewing the young people themselves, their teachers and their parents to understand the nature and extent of the young persons’ friendships and social contact. They found that all children were included in the social networks of their classrooms but to varying degrees. Some children had strong connections to other neurotypical children, while others were on the periphery of social networks. What varied enormously among the children was their motivation for making and keeping friends. While some young people with autism desperately wanted friends, others had limited social connections but preferred things this way: “I am happy with my life right now. I am not friendly and talkative, but I am not not friendly. I am somewhere in the middle” (p. 12).

Calder et al.’s (2013) findings demonstrate that many (though not all) autistic children want to interact with their neurotypical peers, but vary considerably in their motivation to actively engage with them. Intriguingly, all three girls in the sample were strongly motivated to engage with their peers while the remaining boys were less consistent in their desire to do so. This sample is of course small but these data, along with two recent studies (Dean et al. 2014; Head et al. 2014), point towards the possibility that greater sociability and motivation for social contact may be more characteristic of autistic girls than boys.

Consistent with this possibility, Head et al. (2014) found that autistic girls aged 10–16 years scored significantly higher on the Friendship Questionnaire than autistic boys and, furthermore, scored similarly to boys *without* autism. This finding was supported by parental reports of the children’s relationships, suggesting that autistic girls have better social skills and higher social motivation than autistic boys. Similarly, when examining children’s friendship patterns, Dean et al. (2014) showed that autistic boys were more likely to be actively excluded and rejected by their peers, whereas autistic girls were more connected and had higher levels of social motivation, as indexed by a greater number of bids for social interaction during the observation period. Girls with autism also had mostly neurotypical female friends, while boys with autism were generally rejected by neurotypical boys. The authors suggested that the neurotypical friends of autistic girls helped to prevent their active exclusion from social networks, allowing them to maintain their greater connectedness and number of relationships.

Differences in the friendship experiences of autistic boys and girls are perhaps unsurprising, given that it is well known that neurotypical girls and boys have distinct friendship experiences. Among typical girls, for example, friendships are characterised as being more supportive and less characterised by power struggles than those of boys (DeGoede et al. 2009). These differences may be a result of different socialisation patterns. Parents typically encourage gendered play—co-operative pretend play with girls and active physical play with boys—which may have a significant role in later developing friendship patterns (Lindsey and Mize 2001). Furthermore, Barbu et al. (2011) found that typical girls reach more complex social and linguistic development stages earlier than boys, which may allow them to more easily form relationships based on co-operative play and shared conversation.

These gendered patterns of social development might also be true for children on the autism spectrum (see Kreiser and White 2014). Goddard et al. (2014) found that girls on the spectrum have more complex language use when compared to age- and IQ-matched boys on the spectrum. Also, autistic girls tend to have intense interests that revolve around people/animals rather than objects/things and are more similar to those of same-age and gender peers (e.g., celebrities, pop music, drawing) (see Attwood 2006). Their imaginative play also appears to be more gender-typical than that of autistic boys (Knickmeyer et al. 2008; Kopp and Gillberg 1992). Such differences could have knock-on effects for their later interactions with their neurotypical peers, which may make it more likely for girls to be able to engage effectively with their peers.

Understanding any such differences between autistic boys and girls in their social experiences is of critical import. There is emerging consensus from researchers and clinicians that the male preponderance in autism might be overstated—a potential consequence of possible gender-distinct phenotypes and gender inequities in research and diagnostic practices (Goldman 2013; Kreiser and White 2014; Kopp and Gillberg 2011; Lai et al. 2015)—with many girls potentially being misdiagnosed or missing out on a diagnosis until later (Begeer et al. 2012; Giarelli et al. 2010) or even altogether (Dworzynski et al. 2012; see also Gould and Ashton-Smith 2011; Mandy et al. 2011). Knowledge of any differences in the social relationships of autistic boys and girls is therefore crucial for understanding potential phenotypic differences and, if necessary, for developing more refined diagnostic tools and tailored interventions.

This study therefore sought to examine potential gender differences in the social motivation and friendship experiences of adolescent girls and boys with and without autism, which have been hitherto largely unaddressed in the literature. We also focused particularly on adolescents attending specialist educational provision in the UK in part because almost all of the research in this area thus far has been conducted with samples of children who are cognitively able and in mainstream settings.

Children with special educational needs (SEN), which can include a range of developmental conditions, can be at a disadvantage when it comes to their social relationships. They have fewer mutual friends and their friendships are also likely to be less stable than their neurotypical peers, with higher levels of conflict and more issues with repairing the relationship afterwards (Weiner and Schneider 2002). Furthermore, compared to children with SEN in mainstream classrooms, children with SEN in special education settings have fewer friends (Heiman 2000), are less accepted by their peers (Weiner and Tardif 2004), are more frequently bullied (Bunch and Valeo 2004) and exhibit fewer pro-social behaviors (Osborne and Reed 2010).

We focused in particular on adolescents’ motivation for social contact, as measured by the Social Responsiveness Scale (Constantino and Gruber 2012), and the extent and nature of their friendships experiences, as indexed both by a self-completed Friendship Qualities Scale (FQS) (Bukowski et al. 1994) and by an in-depth semi-structured interview on their friends and social contacts. If autistic girls show greater desire for social contact as recent studies suggest, we should expect to find that girls have higher levels of social motivation and qualitatively different friendship experiences than autistic boys. Specifically, the social experiences of adolescent girls with autism should be less like the boys with autism and more like the children without autism (but with additional SEN).

## Methods

### Participants

Forty-six adolescents aged between 12 and 16 years took part in this study, including 13 girls with autism, 13 girls without autism, 10 boys with autism and 10 boys without autism. All participants attended special schools in the south of England and all were in receipt of a Statement of Special Education Needs (SEN), a legal document that details the child’s needs and services that the local education authority has a duty to provide.

All adolescents with autism (n = 23) had received both an independent clinical diagnosis of either autism (n = 19; 10 girls) or Asperger Syndrome (n = 4; 3 girls) according to ICD-10 (WHO 1992) or DSM-IV-TR (APA 2000) criteria and a Statement of SEN, which specified autism as their primary need. Twenty-three participants without autism (13 female; 10 male), but with a range of other difficulties, also participated. These adolescents without autism had a mixture of primary needs as specified in their Statement of SEN, including moderate intellectual disabilities (n = 10; 6 girls), specific language impairment (n = 7; 4 girls), Williams syndrome (n = 1; 1 girl), ADHD (n = 1; girl), and behavioral, emotional and social difficulties^2^ (n = 4; 1 girl). Importantly, none of these 23 participants had an additional clinical diagnosis of autism.

Descriptive information is provided in Table 1. Participants’ IQ scores on the Wechsler Abbreviated Scale of Intelligence (WASI) (Wechsler 1999) fell in the lower end of the normal distribution, in the “extremely low”, “moderately low” and “low average” ranges. Nevertheless, all adolescents had a sufficient level of verbal ability to be able to express their views on their friendship experiences. The four groups were well matched in terms of chronological age, verbal IQ and performance IQ. ANOVAs with group (autistic, non-autistic) and gender (female, male) as between-participant factors revealed no significant main effects of group (*p*s > .18), gender (*p*s > .33) or group × gender interaction (*p*s > .33) for any variable.

**Table 1:** Descriptive statistics for chronological age, Full-Scale IQ, Verbal IQ, Performance IQ, and the Social Responsiveness Scale in boys and girls with and without autism

	Group			
	Girls		Boys	
	With autism (n = 13)	Without autism (n = 13)	With autism (n = 10)	Without autism (n = 10)
Age (years; months)				
M (SD)	14; 0 (1; 1)	14; 0 (0; 11)	13; 10 (1; 0)	13; 6 (1; 1)
Range	12; 4–16; 8	12; 6–15; 1	12; 0–15; 1	12; 0–15; 10
Full scale IQ^a^				
M (SD)	81.17 (11.50)	76.54 (10.25)	78.40 (11.26)	76.54 (10.25)
Range	65–100	62–90	63–98	58–99
Verbal IQ^a^				
M (SD)	77.77 (11.28)	74.08 (8.75)	79.50 (12.14)	74.01 (8.75)
Range	59–98	64–98	66–104	60–84
Performance IQ^a^				
M (SD)	84.00 (15.38)	80.08 (11.32)	81.20 (16.09)	80.08 (14.80)
Range	59–104	62–99	60–103	62–100
SRS-2 total score (scaled)^b^				
M (SD)	72.00 (32.39)	43.00 (13.18)	103.00 (27.76)	40.00 (26.16)
Range	21–129	17–59	64–148	12–97

^a^Children’s intellectual functioning was measured using the Wechsler Abbreviated Scales of Intelligence (WASI; Wechsler 1999)

^b^SRS (Social Responsiveness Scale—2nd edition; Constantino and Gruber 2012)

### Measures

#### Friendship Qualities Scale (FQS)

Adolescents completed Bukowski et al.’s (1994) FQS, which assessed their perceptions of the nature of their relationship with an identified best friend. The scale contains 23 items, which are rated on a 5-point Likert scale ranging from 1 (not true at all) to 5 (very true), and reflect five categories of friendship qualities: (1) Companionship (e.g., “My friend and I spend a lot of our free time together”), (2) Conflict (e.g., “My friend and I disagree about many things”), (3) Help (e.g., “My friend helps me when I am having trouble with something”), (4) Security (e.g., “If I have a problem at school or at home, I can talk to my friend about it”), and (5) Closeness (e.g., “If my friend had to move away I would miss him/her”). Scores on items within each category were summed to yield composite scores measuring each dimension. Higher subscale scores reflect greater friendship quality. The FQS subscales have excellent reliability (Cronbach’s alpha ranges from 0.71 to 0.86). All items are also easily understandable and therefore suited for use with students with SEN.

#### Social Responsiveness Scale—2nd Edition (SRS-2)

Teachers were asked to complete the SRS-2 School-Age Form (Constantino and Gruber 2012), a 65-item rating scale that assesses social and behavioral difficulties associated with autism in children and adolescents. Teachers rate statements about symptoms they have noticed, in the past 6 months, on a 4-point Likert scale ranging from 1 (not true) to 4 (almost always true). It provides scores for five subscales including: (1) Social awareness, (2) Social cognition, (3) Social communication, (4) Social motivation, and (5) Restricted interests and repetitive behavior. Summing scores from individual subscales yields a Social Communication and Interaction (SCI) score and a total raw (SRS) score, which are then transformed to T scores. Higher SRS T-scores reflect greater severity of autistic symptoms. The SRS-2 has excellent reliability (Cronbach’s alpha = 0.95) and strong predictive validity, yielding sensitivity and specificity estimates of 0.92.

#### Semi-structured Interviews

Adolescents were asked a number of open questions about what friendship means to them, the activities they take part in with their friends and their satisfaction with their current friendships (see Calder et al. 2013). Specific items from the “Friends and Marriage” scale of the Autism Diagnostic Observation Schedule—Generic (Lord et al. 2000) were used as a starting point. These items, which were sufficiently broad and open-ended and allowed for children to elaborate, included “Tell me about your friends”, “How often do you see them?” and “What does being a friend mean to you?” We also asked specific questions regarding their motivation to interact with other people such as “Why do you think you are friends with them?”, and “Do you think it is important to have friends at school?” We also investigated young people’s expectations of their friends through two questions asking about different situations: “If you were feeling upset, what would you expect your friend to do?” and “If something nice was happening—like it was your birthday—what would you expect your friend to do?”

All interviews were transcribed verbatim. The resulting data were analyzed using thematic analysis, with particular attention to the phases outlined by Braun and Clarke (2006), including (1) data familiarisation, (2) generation of initial codes, (3) searching for themes, (4) reviewing themes, (5) defining and naming themes, and (6) report production. Two of the authors independently familiarised themselves with the data and met regularly to discuss preliminary themes and codes, to review the results, resolve discrepancies and decide how the codes could be collapsed into themes and subthemes.

### General Procedure

Each participant was seen individually on two occasions, lasting approximately 25–35 min each, at his or her school. In the first session, adolescents completed the WASI. The second session took place approximately 1 week later and included the FQS and semi-structured interview. The length of the interviews ranged from 6.57 to 26.22 min for autistic participants (M = 14.12 min) and 6.34 to 26.32 min for non-autistic participants (M = 14.23 min).

Ethical approval for this study was provided by the University’s Research Ethics Committee. All parents provided informed written consent for their children’s participation and the adolescents themselves also provided written consent to take part.

## Results

**Fig. 1:**
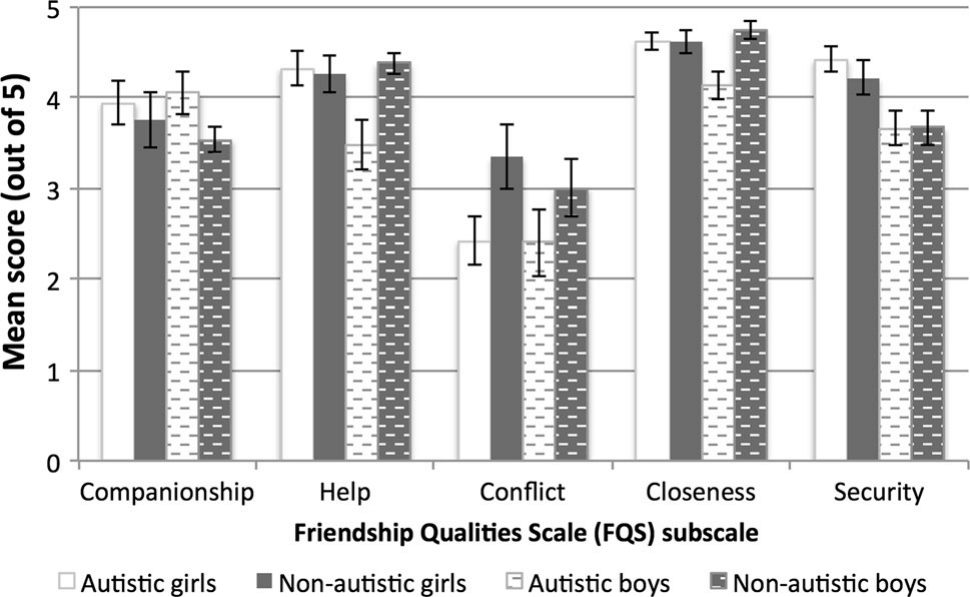
Graph shows adolescents’ mean scores for the Friendship Qualities Scale (FQS) by subscale as a function of gender and diagnostic status. Autistic and non-autistic adolescents are shown in white and grey, respectively (girls: solid bars; boys: patterned bars). Scores on the FQS ranged from 1 (‘not true at all’) to 5 (‘very true’). Error bars represent ± 1 standard error of the mean

This section begins with between-group analyses on participants’ SRS (see Table 1) and FQS scores (see Fig. 1) followed by the results from adolescents’ semi-structured interviews.

### SRS-2

An ANOVA on adolescents’ total (scaled) SRS-2 scores (see Table 1) revealed a main effect of group, F(1, 42) = 36.27, *p* > .001, n_p_^2^ = .46, and a significant group x gender interaction, F(1, 42) = 4.79, *p* = .03, n_p_^2^ = .10, but no main effect of gender, F(1, 42) = 3.80, *p* = .07, n_p_^2^ = .07. Follow-up tests to determine the source of the interaction revealed significant differences between the total SRS-2 scores of adolescent autistic boys and girls, t(21) = .242, *p* = .02, Cohen’s *d* = 1.03, with autistic boys scoring significantly higher than autistic girls. But there were no significant differences between non-autistic boys and girls on total SRS-2 scores, t(21) = .26, *p* = .12 (see Table 1). The small sample size precluded the possibility of examining group and gender differences on all subscales of the SRS-2, but potential differences were examined on the motivation subscale, given that we had a priori reasons to suspect potential differences between autistic boys and girls on this subscale specifically. There was a main effect of group, F(1, 42) = 11.34, *p* = .002, n_p_^2^ = .21, and a significant interaction between gender and group, F(1, 42) = 7.45, *p* = .009, n_p_^2^ = .15. There was no main effect of gender, F(1, 42) = .42, *p* = .52. Further between-group analyses revealed no significant differences between non-autistic boys (M = 6.40; SD = 4.93) and girls (M = 9.92; SD = 5.02) on the social motivation subscale, t(21) = 1.68, *p* = .11, but autistic boys had significantly higher scores (reflecting lower social motivation; M = 16.70; SD = 6.91) than autistic girls (M = 11.0; SD = 5.80), t(21) = 2.15, *p* = .04, *d* = 0.89. Furthermore, while autistic boys obtained significantly higher scores than non-autistic boys, t(18) = 3.84, *p* = .001, *d* = 1.72, girls with and without autism did not score significantly different on this subscale, t(24) = .51, *p* = .62.

### FQS

ANOVAs were conducted to examine group and gender differences for each FQS subscale separately (see Fig. 1). For the Companionship subscale, there were no group or gender differences or any group x gender interaction (all *p*s > .28). Girls and boys with and without autism appear to perceive their friends in a similar way in this regard.

On the Help subscale, there was a significant main effect of group, F(1, 42) = 4.78, *p* = .03, n_p_^2^ = .10, and a significant group x gender interaction, F(1, 42) = 6.21, *p* = .01, n_p_^2^ = .13. There was no main effect of gender, F(1, 42) = 2.90, *p* = .10, n_p_^2^ = .06. Autistic boys’ Help subscale scores were significantly lower (reflecting fewer helping behaviors) than autistic girls, t(21) = 2.65, *p* = .01, *d* = 1.10, and non-autistic girls and boys (both *p*s < .01). There were no significant differences between autistic girls and adolescents (boys or girls) without autism (*p*s > .82).

A similar pattern was found for the Closeness subscale. An ANOVA on adolescents’ Closeness scores revealed a main effect of group, F(1, 42) = 6.28, *p* = .01, n_p_^2^ = .13, a significant interaction between gender and group, F(1, 42) = 6.28, *p* = .01, n_p_^2^ = .13, but no effect of gender, F(1, 42) = 2.15, *p* = .15, n_p_^2^ = .05. Follow-up *t* tests showed that autistic boys reported less intimacy in their best-friendships than autistic girls, t(21) = 2.81, *p* = .01, *d* = 1.15, and than non-autistic adolescents (*p*s < .005). There were no other significant differences between groups (*p*s > .42).

Analysis of adolescents’ scores on the Security subscale revealed a main effect of gender, F(1, 42) = 14.14, *p* = .001, n_p_^2^ = .25, but no effect of group (*p* = .51) or gender x group interaction (*p* = .66). Boys (with and without autism; M = 3.64, SD = .59) generally reported lower scores on the Security items, suggesting that they perceived their best-friendships as less secure than girls (with and without autism; M = 4.32, SD = .60).

On the Conflict subscale, there was a significant main effect of group, F(1, 42) = 5.41, *p* = .02, n_p_^2^ = .11, but no effect of gender or interaction involving gender (both *p*s > .60). Autistic adolescents reported significantly lower scores on the Conflict items (reflecting a perceived lack of conflict in their relationships; M = 2.41, SD = 1.03) than non-autistic adolescents (M = 3.21, SD = 1.16).

To summarise, autistic girls reported the quality of their friendships to be similar in nature to non-autistic girls (in terms of Companionship, Help, Closeness and Security) with the exception of lower levels of Conflict in their friendships. Autistic boys reported their friendships to be qualitatively different, reflected by lower ratings on the Help, Closeness and Conflict items, from non-autistic boys. Non-autistic boys and girls only differed in terms of Security, with non-autistic boys perceiving their friendships as less secure than non-autistic girls. Autistic boys and girls, however, differed considerably in terms of their perceived friendships: autistic girls reported their friendships to be closer, more helpful and more secure than autistic boys.

### Semi-structured Interviews

During the interviews, all participants named at least one ‘best’ friend in school, although they often talked about multiple friends. Two participants were the exception to this pattern, one naming a neighbour and the other, a sibling, as their best friends. Nearly all adolescents stated that they saw their friends outside of school, albeit infrequently, and elaborated that they would like to be able to meet them more often or to spend time with friends they currently saw only in school. For many participants, practicalities prevented spending more time with friends outside school—for example, living far away from each other. Almost all adolescents felt that it was important to have friends in school. Two autistic participants—both boys—did not feel this way and expressed ambivalence towards having or needing friends. Also, while all non-autistic participants were content with the number of friends they already had, a minority (n = 5; 2 girls) of the autistic participants felt that they would like more friends.

Three main themes were identified in adolescents’ descriptions of their friendships, including Companionship (including three sub-themes: friends are people to hang out with, friends make me laugh, and ‘girl talk’); Scripting (saying what you’re supposed to); and Conflict (when things get tough). Participants’ quotes are identified by their group membership (NB: non-autistic boy; NG: non-autistic girl; AB: autistic boy; AG: autistic girl).

#### Companionship: Friends are People to Hang Out with

Companionship emerged as the dominant theme across all interviews, as characteristics associated with it were consistently presented as the definition of ‘a friend’: “good people to play with” (NB), “my friends like hanging around with me” (AB), “they would always look after me” (AG) and “they’re fun” (NG). This focus on the active aspects of friendship was evident across all participants, although there was variation in the types of activities in which they engaged with friends. Boys were more likely to talk about games and doing the same things as their friends—“play UNO” (AB), “play football or play manhunt” (NB)—and even noted that not having the same interests could be a barrier to being friends: “not all of us have the same hobbies … we can sometimes get on each others’ nerves” (AB). This was similar for both non-autistic and autistic boys, suggesting commonalities in the nature of their friendships.

All participants discussed the need for companionship as their major form of social motivation. The idea of being alone at break or lunchtime generated a negative response from most participants: “who would you sit around with at lunch or who would you hang around with?” (AG); “so I can get a bit of company at break-time … so you’re not on your own all the time” (AG); “some people are lonely and need friends” (NG). Two autistic boys, however, talked about this issue either in a detached way (“if they’re there, they’re there, and if they’re not, they’re not”; AB) or expressed that they would rather be on their own at these times as a way of getting some quiet time (“I stand near the staff room … because the playground is stupid”; AB).

#### Companionship: Friends Make Me Laugh

Being able to share humour with their friends was given a high profile for adolescents with *and* without autism. ‘Being funny’ was given as a key characteristic of a friend (“they tell funny jokes”, AB; “they make me laugh a lot”, AG), with many coming back to humour repeatedly as an indicator of whether this was a ‘good’ friendship (e.g., “she’s got the same sort of things as me. Like laughing. We giggle a lot”, NG; “They have to be funny. Definitely”, AG). Humour was also often used to identify the adolescents’ friendships as ‘normal’—“it’s just normal things for friends to do and it’s just funny” (AB), “no problem, just up for having a laugh” (AG) in contrast to elements of their lives which were not, such as having an “escort” [i.e., teaching assistant] (NB).

#### Companionship: ‘Girl Talk’

Girls’ descriptions of friendship focussed on shared talk significantly more than shared activities, an element which was absent in the boys’ explicit descriptions. Both autistic and non-autistic girls followed this pattern, identifying it as a significant element in their friendships: “Being a friend means you have someone to talk to” (AG); “we just hang around, and get to, like, talk and get to know each other” (NG). Characteristically feminine topics of conversation (e.g., “boys can come into the subject”, NG; “gossiping … talk about fashion, clothes, prize giving”, AG; “girly stuff … boys and stuff and teenage stuff and gossip”, AG) were also mentioned by most of the female participants. These topics of conversation also reflect a focus on the relationships between *people*, in contrast to the boys’ conversational focus on *actions* or *objects* (“we normally laugh about other people’s food”, NB).

#### Scripting: Saying what You’re Supposed to Say

Adolescents’ responses to certain questions often followed a standard pattern and were thus perceived to be ‘scripted’, as if they were echoing something that they had heard before. For example, many adolescents used adult phrases when describing friends (e.g., “happy old chaps”, NB). Importantly, such scripted responses were observed across all interviews, although the most obviously scripted answers were from autistic boys and the least scripted from non-autistic girls. Autistic girls and non-autistic boys had similar levels of scripting, although autistic girls used scripting more in relation to emotional expectations and included phrases such as “say “Don’t cry” and stuff” (AG) or “say it’s alright and stuff like that” (AG). Non-autistic boys used more action-based scripting than autistic girls, such as about expected responses to something nice happening, like the participant’s birthday: “give me a birthday card” (NB) compared to “be nice to me” (AG), or “be happy” (AG).

Some autistic girls did provide action-based responses (e.g., “bring presents”; AG) and some non-autistic boys did provide emotion-based responses (e.g. “congratulate you or be nice”, NB). The majority of non-autistic girls, however, gave answers based on shared emotions in response to both scenarios (e.g., “come over and ask me what’s the matter”; NG, “I would expect my friend to be happy [for me]”; NG).

#### Conflict: When Things Get Tough

There was a marked discrepancy between the reported levels of conflict in autistic adolescents’ relationships on the FQS and the extent to which they discussed conflict in the interviews. This was particularly true for autistic girls who rated their relationships as having less conflict than non-autistic girls, but who discussed a wide range of often-aggressive incidents. The relationally aggressive behaviors characteristic of many typical female adolescent friendships (see Nichols et al. 2009) such as gossiping, being excluded, and having trust betrayed were discussed repeatedly by the autistic girls: “she may ignore me” (AG); “D a few weeks ago tried to take A away from us” (AG); “basically just backstabbing, bitchin’ … people go and say something to one people and the other person goes around and tells another person” (AG) and non-autistic girls: when someone “tells your secret” (NG) or “saying that they had done something when they really haven’t” (NG). Such behaviors featured less often in all boys’ interviews.

It is worth noting that the autistic girls who described these incidents did not see their friendships overall as being characterised in this way. The examples given were again based on behaviors linked to relational conflict: “getting people upset” (AG) or “if they go and play with somebody else” (AG), but they often said that their friends had not annoyed them or that there were very few ‘not-so-good’ things about their friendships.

## Discussion

This study investigated the social motivation and friendship experiences of adolescent boys and girls, with and without autism, in special education settings. Teachers reported that the autistic girls in their classes had less severe social difficulties than the autistic boys. In fact, autistic boys stood out as having significantly lower levels of social motivation, and appeared to have qualitatively different friendships, than all other groups. In contrast, autistic girls rated their friendships similarly to non-autistic girls on all FQS subscales except the Conflict dimension, on which they reported lower levels than non-autistic girls. This pattern of findings was corroborated by adolescents’ descriptions of their friendship experiences, with the exception of the degree of conflict in autistic girls’ relationships, who reported greater levels of conflict than their questionnaire responses initially suggested.

One aim of this study was to examine potential gender differences in the degree of social motivation in adolescents with autism. Here, we showed that girls with autism had greater social motivation—as demonstrated by their higher SRS subscale scores and greater discussion of engaging with other people in the interviews—than autistic boys. In fact, boys with autism expressed less concern with making and maintaining friendships in school than autistic girls, sometimes reporting wanting to avoid social interactions. This reduced social motivation is consistent with results from Whitehouse et al. (2009), which showed that their group of adolescents (mostly boys) with Asperger’s syndrome reported lower scores on Richard and Schneider’s (2005) Friendship Motivation Questionnaire, suggestive of less self-determined motivation for friendships. Furthermore, the boys’ comments in the current study were similar to those made by children with autism in Calder et al. (2013), one of whom stated, “sometimes I just want to play by myself” (p. 12), suggesting that the opportunities to be away from others can be just as important as being with them (see also Humphrey and Lewis 2008).

The girls with autism in our study, however, were rated by their teachers as having greater levels of social motivation relative to boys with autism, and similar such levels relative to the non-autistic adolescents. Furthermore, unlike boys with autism, the descriptions of autistic girls’ friendships were centred more on people rather than actions or objects, again suggestive of a greater interest in social contact. Head et al. (2014) also reported that autistic girls showed greater interest in the relationships of other people, as well as in their own direct relationships with others, compared with autistic boys.

Together, these findings suggest key differences in the sociability of adolescent boys and girls on the autism spectrum. They also raise questions regarding the underlying nature of such differences. Some authors suggest that social motivation, which drives human behavior, is fundamentally diminished in autism (Chevallier et al. 2012). Our results, however, clearly indicate that such an explanation cannot be applicable to all individuals on the autism spectrum—especially adolescent girls. Indeed, one recent study found that more parents reported that their young autistic girls were able to engage in complex imitation (e.g., imitation games or multiple actions) than parents of autistic boys (Hiller et al. 2015). Such prowess in autistic girls’ imitation skills could be one early manifestation of these girls’ later social interest and motivation to engage with others. The cause of this apparent gender-dependent characteristic is unclear, although culture-based gender role expectations (of parents, peers, broader society) related to social sensitivity and emotional attunement are likely to play an important role in shaping the way that social (dis)abilities are manifested in girls on the autism spectrum (see Goldman 2013; Kreiser and White 2014, for discussion).

This possibility is further supported by our finding of gender-dependent differences in the nature of autistic adolescents’ friendship experiences. We found significant differences between autistic boys and their non-autistic peers (boys and girls), with autistic boys rating their best-friendships as less close, less secure, and having less conflict and lower levels of helping behaviors. These findings replicate those of existing studies (Calder et al. 2013; Locke et al. 2010) and extend them to boys with additional intellectual and learning needs, echoing previous research findings about the friendships of children with other learning disabilities (Weiner and Schneider 2002; see Webster and Carter 2014, for review). Furthermore, and importantly, we showed that the friendship experiences of boys and girls on the autism spectrum were qualitatively distinct. While all adolescents reported companionship as a key quality of their friendships, autistic boys rated their friendships as containing less intimacy than autistic girls (and non-autistic adolescents) and also described their friends in less affective ways. These findings resonate with work reporting that autistic children (mostly boys) are more likely to focus on ‘active’ rather than ‘affective’ components of relationships (Bauminger and Kasari 2000). They also support one of the few existing studies in this area, which found that autistic girls showed different friendship patterns to autistic boys, such that they were more included in classroom social networks with their neurotypical same-gender peers (Dean et al. 2014).

Critically, however, these findings also highlight that autistic girls’ perceptions of their friendships were more similar to those of the non-autistic girls and boys than the boys with autism. Autistic girls had very similar scores on the FQS for the majority of friendship dimensions, and were just as likely to partake in “girl talk”—conversation focused on stereotypically female interests such as boys, fashion and shopping—as the non-autistic girls. While the underlying causes for these behaviors are unclear, these findings, if replicated, nevertheless have significant implications for identifying autism in girls. Although the DSM-5 (APA 2013) acknowledges that gender differences probably exist in autism, they provide no specific descriptions of how such differences might manifest behaviorally. The current data clearly show that the degree of sociability and nature of social relationships might be qualitatively different in boys and girls with autism—at least during adolescence—and might be one reason why girls tend to be clinically identified later than their male counterparts (Begeer et al. 2012; Giarelli et al. 2010) or why they might slip ‘under the radar’ all together (Dworzynski et al. 2012; Russell et al. 2011).

Girls with autism were not completely free of social difficulties, however. One key difference between girls with and without autism related to the extent and nature of conflict experienced in their friendships. Girls with autism reported significantly less conflict in their best-friendships on the FQS than girls without autism but nevertheless discussed many instances of what can be termed as ‘relational conflict’ (Nichols et al. 2009), including gossiping, interfering in relationships, excluding individuals socially and ‘stealing’ friends. The very presence of relational aggression acts within both autistic *and* non-autistic girls’ friendships emphasises the possibility that these girls’ friendships may on the whole be more similar to each other than to boys with or without autism. Nevertheless, this discrepancy between autistic girls’ quantitative and qualitative data *and* their apparent lack of understanding of this conflict in the interviews suggest that they might not necessarily be able to recognise conflict in their relationships and/or be able to manage such conflict in the same way as non-autistic girls. Although girls’ greater interest in others might enable them to initiate social contact and make friends with others, core social and communication difficulties could mean both that they struggle to respond to subtle social nuances (Dean et al. 2013) and that they are an ‘easy target’ for relational conflict—all of which could contribute to their greater susceptibility of being ‘socially neglected’ rather than actively rejected in the same way as some autistic boys (Dean et al. 2014).

## Conclusions

This is the first study to investigate the gender differences in the social relationships of adolescents with autism in special education settings. Overall, girls with autism were more socially motivated and reported friendships that were more intimate than those of boys with autism—which meant that their social experiences were more similar to those of non-autistic boys and girls than to autistic boys. The exception to this pattern lay in their understanding of conflict within their relationships. This novel finding warrants further investigation, especially since it could make an important target for intervention to ensure that autistic girls are able to both obtain *and* sustain social relationships.

Although the sample size was small, confidence in the findings is warranted given their parity with previous findings (e.g., Head et al. 2014) and the broad consistency across questionnaire and interview methods. One potential limitation of the study is the inclusion of adolescents in the non-autistic group with a wide variety of developmental conditions (other than autism). These different conditions are likely to yield different social and developmental outcomes for each child, which may have increased the variation within the non-autistic groups seen here. The clear similarities and differences between the groups of adolescents with and without autism, however, suggest that the effects of a heterogeneous sample may be limited. Nevertheless, future, more well-powered studies should seek to determine further the specificity of the effects reported here—particularly with regards to difficulties identifying and managing conflict in autistic girls—by comparing directly groups of boys and girls with autism with more homogenous groups of boys and girls (e.g., adolescents with ADHD or specific language impairment). Future work should also seek to determine whether the reported gender differences extend to cognitively able adolescent boys and girls with and without autism who are educated in mainstream settings, and to understand the extent to which qualitative differences in social motivation and friendship have on the wellbeing and mental health of young autistic people—boys and girls—in the long term.

## Footnotes

1 The term ‘autistic person’ is the preferred language of many people on the spectrum (see Sinclair 1999; Kenny et al. 2015). In this article, we use this term as well as person-first language to respect the wishes of all individuals on the spectrum.

2 In the recent SEN reforms (Department for Education 2014), the category of “behavioral, emotional and social difficulties’” (BESD) has been replaced by a new term, “social emotional and mental health” (SEMH), to reflect better the underlying causes of students’ behavioral difficulties.
